# Performance Analysis of Surface Reconstruction Algorithms in Vertical Scanning Interferometry Based on Coherence Envelope Detection

**DOI:** 10.3390/mi12020164

**Published:** 2021-02-08

**Authors:** Dongxu Wu, Fusheng Liang, Chengwei Kang, Fengzhou Fang

**Affiliations:** 1 Centre of Micro/Nano Manufacturing Technology (MNMT-Dublin), University College Dublin, Dublin 4, Ireland; dongxu.wu@ucd.ie (D.W.); fusheng.liang@ucd.ie (F.L.); chengwei.kang@ucd.ie (C.K.); 2 School of Control Engineering, Northeastern University at Qinhuangdao, Qinhuangdao 066004, China; 3 State Key Laboratory of Precision Measuring Technology and Instruments, Centre of Micro/Nano Manufacturing Technology (MNMT), Tianjin University, Tianjin 300072, China

**Keywords:** vertical scanning interferometry, algorithm, low coherence, envelope detection, surface topography, height measurement

## Abstract

Optical interferometry plays an important role in the topographical surface measurement and characterization in precision/ultra-precision manufacturing. An appropriate surface reconstruction algorithm is essential in obtaining accurate topography information from the digitized interferograms. However, the performance of a surface reconstruction algorithm in interferometric measurements is influenced by environmental disturbances and system noise. This paper presents a comparative analysis of three algorithms commonly used for coherence envelope detection in vertical scanning interferometry, including the centroid method, fast Fourier transform (FFT), and Hilbert transform (HT). Numerical analysis and experimental studies were carried out to evaluate the performance of different envelope detection algorithms in terms of measurement accuracy, speed, and noise resistance. Step height standards were measured using a developed interferometer and the step profiles were reconstructed by different algorithms. The results show that the centroid method has a higher measurement speed than the FFT and HT methods, but it can only provide acceptable measurement accuracy at a low noise level. The FFT and HT methods outperform the centroid method in terms of noise immunity and measurement accuracy. Even if the FFT and HT methods provide similar measurement accuracy, the HT method has a superior measurement speed compared to the FFT method.

## 1. Introduction

Optical interferometry is widely regarded as a powerful tool for topographical surface metrology due to its significant advantages of non-contact operation, high accuracy, and high resolution [1,2,3]. It has been gaining great importance for the manufacturing of high value-added components, such as freeform optical elements [4,5,6,7], micro-electro-mechanical-systems (MEMS) [8], microstructures [9,10], and transparent thin films [11]. Among the developed interferometric techniques, phase-shifting interferometry (PSI) is generally renowned for measurement precision as high as λ/1000 but it suffers from the 2π ambiguity problem, requiring that the surfaces to be measured should be smooth and continuous [12]. Vertical scanning interferometry (VSI) (also known as coherence scanning interferometry (CSI) [13]) is an effective means for performing three-dimensional (3D) areal topography measurements. In contrast to PSI, the principle of VSI is that the interference fringe contrast is monitored corresponding to the zero optical path difference (OPD), rather than the underlying phase distribution. The fringe visibility of a low coherence interferogram produced in VSI is narrowly localized in the spatial domain, which contributes to the unambiguous determination of the OPD between the test and reference beams [14]. Therefore, VSI effectively extends the applications of interferometric techniques in surface measurement and characterization, being suitable for more surface textures in terms of roughness, slopes, discontinuities, and structures [15].

Digitized fringe processing is essential to surface topography reconstruction in interferometric measurements. Nowadays, a variety of fringe analysis algorithms have been developed to meet various measurement requirements. Surface topography information can be estimated from the coherence envelope and the phase of interference fringes [14]. The centroid approach has been used to identify the central fringe order and estimate the modulation peak in broad bandwidth interferometry [16,17]. Finding the centroid of an interference pattern is a simple and computationally economical method, which is especially suitable for rapid surface measurement. However, the centroid of the coherence envelope function may not be necessarily coincident with the envelope peak. If the amplitude-modulated interferometric signal is not completely symmetrical due to environmental disturbances and system errors, there would be an offset between the calculated centroid and the peak position corresponding to the maximum fringe contrast, resulting in a large measurement error [18]. The fast Fourier transform (FFT) was employed by Chim and Kino [19] to extract amplitude and phase information from the interferograms collected in a Mirau correlation microscope. The FFT method has enjoyed tremendous popularity in envelope detection and phase demodulation due to its notable advantages such as only one (or two) fringe(s) needed, full-field analysis, and high precision [20,21]. However, FFT-based algorithms are time-consuming because the fringe contrast for each point needs to be calculated by performing one forward transform and one inverse transform. Wei et al. [22] proposed that only the frequency pairs with a high signal-to-noise ratio (SNR) were needed for envelope peak determination, but the proposed method was not applicable in an environment with fluctuating noise. Hence, noise reduction methods in practical measurements are of great importance to VSI and are still attracting wide attention [23].

The Hilbert transform (HT) is an alternative method to obtain coherence envelope and phase values from the captured interference signals [24,25,26]. This method effectively reduces the computational complexity and allows a faster processing speed for real-time imaging [27]. Pavliček and Michálek [28] revealed the effect of noise on the envelope detection and measurement uncertainty of white light interferometry by HT. To improve noise resistance, Trusiak et al. [29] used the Hilbert–Huang transform (HHT) to develop a demodulation algorithm that was robust to fringe pattern imperfections and environmental disturbances. Gianto et al. [30] made a comparison of envelope detection techniques and proposed a compact and robust algorithm by using the Teager–Kaiser energy operator. This improved algorithm is faster than the continuous wavelet transform and more competitive than the HT and five-sample adaptive methods in terms of surface extraction. It should be noted that the HT-based algorithms can yield accurate measurement results only when the background intensity in a captured interferometric signal is constant. However, it is challenging to precisely eliminate the background term from the interferograms in practical measurements.

In contrast to phase-shift methods, spatial phase-demodulation methods, such as FT and HT, offer better resistance to noise because phase extraction at each pixel is affected by its neighboring pixels or even all pixels in the fringe pattern [31]. However, one typical weakness of spatial fringe analysis techniques is that the diffraction effects due to surface discontinuity may modify the coherence envelope and cause its peak to shift. A solution to the above weakness is to combine the strengths of VSI and PSI. Specifically, Larkin [32] investigated the suitability of phase-shifting algorithms for envelope detection of white-light interferograms and demonstrated the optimal computational efficiency of the derived algorithm. Harasaki et al. [12] improved the height resolution and removed the bat-wing effect of VSI by comparing the two profiles obtained from the coherence-peak-sensing technique and phase measurement at the best-focus frame position, respectively. To accurately locate the coherence peak position, Vo et al. [33] proposed a novel algorithm that combined the white-light phase-shifting interferometry (WLPSI) method with the coherence-peak-sensing technique. The combined WLPSI algorithms can effectively improve the measurement accuracy and offer a large dynamic range without a 2π ambiguity problem, but the trade-off is computational load and processing time. Kiselev et al. [34] proposed a correlogram correlation method that used the covariance of a correlogram measured with the reference correlogram at the best fitting position as the criterion for the appropriateness analysis, providing fewer outliers than the envelope parabola method when measuring a rough groove wall of about 40° pitch.

Over the past few decades, tremendous efforts have been made to develop novel fringe analysis algorithms with the desired features. The selection of an appropriate algorithm for a specific requirement should be made by choosing the one with the optimal strengths and least weaknesses for the given task. Furthermore, there is a strong demand for on-machine or even in-process measurement for ultra-precision manufacturing [35,36,37]. In this case, the measurement performance of a VSI system is affected by many influence factors. The accuracy, calculation efficiency, and robustness of the selected algorithm need to be comprehensively evaluated. To address this issue, this paper presents a comparative analysis of surface reconstruction algorithms based on coherence envelope detection. Firstly, the principle of VSI and the theoretical background of three kinds of fringe analysis algorithms based on the centroid, FFT, and HT methods are discussed. A series of computational simulations are carried out to investigate the effects of random noise and scanning interval on white-light interferogram analysis. Furthermore, some experiments are conducted on a miniaturized white-light interferometer developed for on-machine surface measurement. Finally, the performance of different algorithms on step height measurement is comparatively studied. This paper contributes to the understanding of fringe analysis algorithms based on envelope detection techniques.

## 2. Principle and Method

### 2.1. Vertical Scanning Interferometry

**Figure 1:**
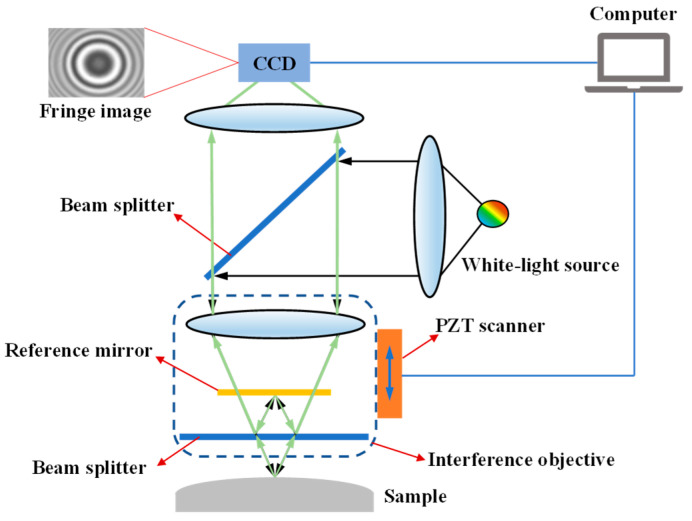
Schematic of the vertical scanning interferometry (VSI) system.

A VSI system equipped with a Mirau-type objective is illustrated in Figure 1, which mainly consists of a white-light source, interference objective, piezoelectric transducer (PZT) scanner, and charge-coupled device (CCD) camera. The beam provided by a white-light source is reflected by the upper beam splitter and directed into the interference objective. Two optical paths, including a reference path and a measuring path, are produced when the beam goes through the beam splitter into the interference objective. The beam reflected by the sample surface recombines with the beam reflected by the reference mirror, allowing the generation of interference fringes [38]. The interference objective is driven by a computer-controlled PZT scanner, resulting in variations in OPD between the measuring beam and reference beam during a continuous vertical scanning motion. Thus, a series of contrast-modulated interference fringes are generated and detected by the CCD camera. Due to the low coherence property of the spectrally broadband light, the maximum fringe contrast occurs only when the test surface has zero OPD with respect to the reference surface. VSI is capable of determining the zero-order fringe without introducing a phase ambiguity problem, thus offering a larger dynamic measurement range.

**Figure 2:**
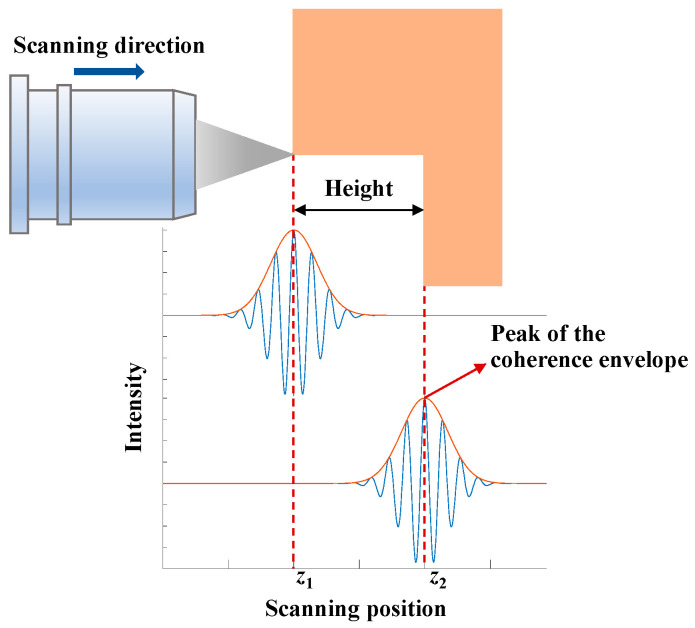
Illustration of data acquisition in VSI.

The working principle of VSI is to seek the scanning position of the coherence envelope peak. As shown in Figure 2, the objective moves along the vertical scanning direction and focuses on the upper and lower surfaces of the step, respectively. The scanning positions of the PZT and the interference intensity are synchronously recorded by the computer for each pixel in the CCD camera. The overall contrast feature of the interference pattern, such as the centroid or peak of the coherence envelope, is evaluated and the envelope peak position for each pixel can be extracted to determine the height information [39].

### 2.2. Fringe Analysis Algorithm

The fringe analysis algorithm plays an important role in surface topography reconstruction. Algorithms based on coherence envelope detection are employed to estimate the envelope center of the interference fringe, rather than the phase information of the central wavelength. Surface heights under test can be directly obtained by finding the centroid or peak position of the coherence envelope.

#### 2.2.1. Centroid Method

When the VSI system is illuminated by a broadband light source, the fringe contrast decreases sharply with the distance away from the position of zero OPD. An amplitude-modulated interferometric signal is approximately symmetrical at zero OPD, in which the modulation component can be represented by a bell-shaped function. Thus, the peak position of the coherence envelope can be estimated directly by finding the centroid of the modulation function. During discrete scanning measurements, the light intensity *I*(*z*) of an interferometric signal and the vertical position corresponding to the centroid can be respectively expressed as [17]

(1) $$I(z)=I_{0}+m(z)cos(\omega_{0}z+\varphi_{0}),$$

(2) $$z=\frac{\sum \left[zf(z)\right]}{\sum f(z)},$$

where *z* is the vertical scanning position, *I*_0_ is the background intensity, *m*(*z*) refers to the modulating signal that approximates a symmetrical bell-shaped function *f*(*z*), *ω*_0_ and *φ*_0_ are the fringe signal and initial phase, respectively. Hence, the height data at each pixel can be determined rapidly by the centroid position of the whole interference pattern. For an asymmetric amplitude-modulated interferometric signal, the quadratic functions of its first-order derivative are convergent and have a similar centroid with the modulation function. A more desired centroid close to the modulation peak position can thus be given by [17]

(3) $$z=\frac{\sum \left\{z{\left[I^{\prime }(z)\right]}^{2}\right\}}{\sum {\left[I^{\prime }(z)\right]}^{2}},$$

where $I^{\prime }(z)$ is the first-order derivative of the interferometric signal. Centroid determination for the whole interference pattern is a simple and computationally economical method, thus being capable of rapid surface topography measurement.

#### 2.2.2. Fourier Transform Method

The normalized frequency spectrum of a broadband light source is approximately equal to a Gaussian function. The white-light interferogram has a distinct feature in that it is a cosine-modulated function [40]. The output light intensity from a CCD camera is due to the cross correlation between the measuring signal *A*(*x*, *y*) and the reference signal *B*. After removing the background bias (*A*^2^(*x*, *y*) + *B*^2^), the correlation term of the interferometric signal for each pixel (*x*, *y*) on the sample can be expressed as [19]

(4) $$I_{AB}=Cg\left[z-z_{0}(x,y)\right]\cos\left\{\varphi \left[\left(z-z_{0}(x,y\right)\right]\right\},$$

where *C* is twice the product of *A*(*x*, *y*) and *B*, $g\left[z-z_{0}(x,y)\right]$ is an envelope function that modulates the correlation term, and $\varphi \left[\left(z-z_{0}(x,y\right)\right]$ refers to the phase. The envelope peak of *I_AB_* can be found at $z=z_{0}(x,y)$. A forward FFT is performed along the vertical scanning direction for all pixels. After filtering the negative-frequency components and centering the packet of the positive-frequency components in the Fourier domain, an inverse FFT is carried out to obtain the coherence envelope. The demodulated correlation function can be written as [19]

(5) $$I_{AB}^{d}=Cg\left[z-z_{0}(x,y)\right]\exp\left\{i\varphi \left[z_{0}(x,y)\right]\right\},$$

Therefore, the surface height can be determined by extracting the envelope peak of $I_{AB}^{d}$. The main concern in FFT-based algorithms is the computational intensity due to its global property.

#### 2.2.3. Hilbert Transform Method

Similar to FFT, HT works by extracting the low-frequency components of the interferometric signal while filtering the high-frequency component [30]. As illustrated in Equations (1) and (4), the light intensity of the interferometric signal at a given point varies with the vertical scanning position *z* and has a cosine phase variation. A complex analytic signal $V_{xy}(n)$ that represents the demodulated correlation function described in Equation (5) can be constructed as [24]

(6) $$V_{xy}(n)=i_{xy}(n)+ji_{xy}*(n),$$

where $i_{xy}(n)$ is an unbiased intensity, $i_{xy}*(n)$ refers to π/2 phase-shifted $i_{xy}(n)$. The phase-shifted $i_{xy}*(n)$ can be obtained by convolving $i_{xy}(n)$ with a normalized impulse response of HT with (2*M* + 1) elements. Thus, the phase-shifted $i_{xy}*(n)$ can be simplified to the form [24]

(7) $$i_{xy}*(n)=\left\{ \begin{array}{ll} \frac{2}{\pi }\sum_{m=1}^{M}\frac{i_{xy}(m-n)-i_{xy}(m+n)}{m} & \mathrm{for}\;m\;\mathrm{is}\;\mathrm{odd}\\ 0 & \mathrm{otherwise} \end{array}\right.,$$

Based on the known $i_{xy}*(n)$, the modulus of the analytic signal $V_{xy}(n)$ that corresponds to the envelope of the interferometric signal can be obtained.

## 3. Simulation Results and Discussion

To investigate the measurement performance of the algorithms described above, simulation studies were carried out in white-light interferogram analysis. As previously discussed, the maximum fringe contrast occurs only when the OPD between the reference beam and the measuring beam is zero. The surface heights under test can be determined by seeking the envelope peak position where there exists a maximum fringe contrast. Based on Equation (1), the light intensity of a point in the white light interferogram can also be expressed as [33,38]

(8) $$I\left(z\right)=I_{0}+\gamma I_{0}\exp\left[-{\left(\frac{z-z_{0}}{l_{c}}\right)}^{2}\right]\cos\left[\frac{4\pi }{\lambda_{0}}\left(z-z_{0}\right)+\varphi_{0}\right]$$

where *γ* is the fringe contrast, *z*_0_ is the peak position of the zero-order fringe, *l*_c_ is the coherence length of the light source, and *λ*_0_ is the central wavelength. To generate a set of simulated white light interferometric signals for *I*_B_ = 400, *γ* = 1, *λ*_0_ = 550 nm, *φ*_0_ = 0 rad, Equation (8) can be rewritten as [33]

(9) $$I_{N}=400+400\exp\left[-\frac{{\left(N-1000\right)}^{2}\Delta^{2}}{\sigma^{2}}\right]\cos\left[\frac{4\pi }{550}\left(N-1000\right)\Delta \right],$$

where *N* is the step number, *σ* = *l*_c_/2π and is set to be 500 nm, and Δ indicates the scanning interval. The peak position of the coherence envelope can be obtained at *N* = 1000, corresponding to 30 μm surface height for a scanning interval of 30 nm. The computation processes were performed on a commercial laptop (i7-8550U CPU, 8.00 GB RAM, NVIDIA GeForce MX150 graphics card). The algorithms were implemented using a Matlab program.

### 3.1. Effect of Random Noise

**Figure 3:**
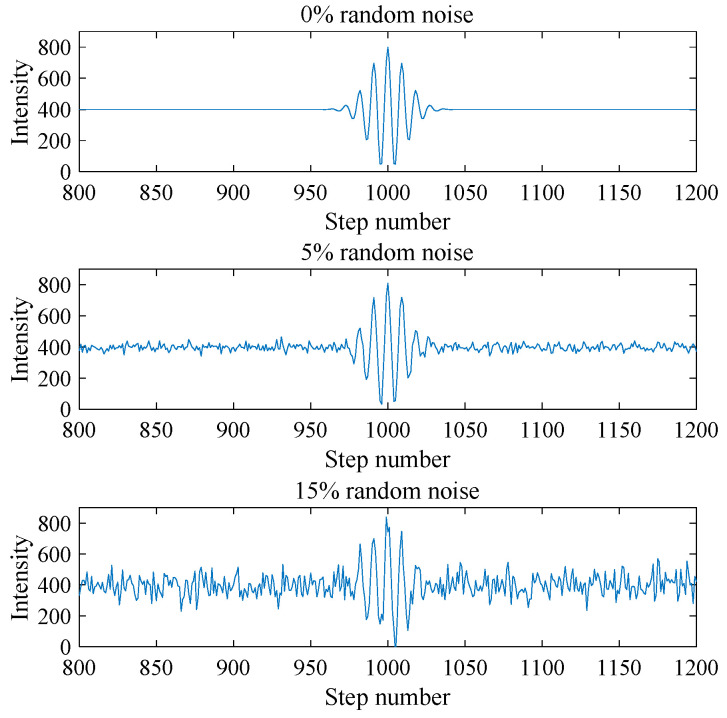
Simulated white light interferometric signals under different noise levels.

To investigate the noise immunity of different surface reconstruction algorithms, the ideal white-light interferometric signal was superimposed with random Gaussian noise. As shown in Figure 3, a series of white light interferometric signals are generated with random noise at the level of 0%, 5%, and 15%. When no noise is added, the ideal interferometric signal is smooth and symmetrical. As the random noise increases, the interferometric signal begins to be rough and the zero-order fringe peak seems to shift.

**Figure 4:**
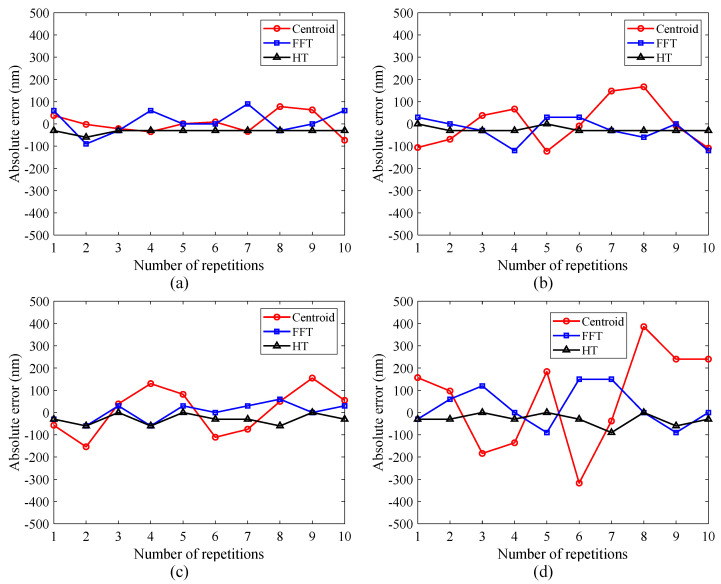
Simulation results of the interferometric signal under different noise levels: (**a**) 5% random noise, (**b**) 10% random noise, (**c**) 15% random noise, and (**d**) 20% random noise.

Under different noise levels incorporated by the simulated interferometric signals, the surface heights were calculated 10 times by using different algorithms. Figure 4 shows a comparison of absolute height errors in simulation. As shown in Figure 4a, with a 5% random noise added, there is a slight change in the absolute height errors introduced by the centroid and FFT methods, while the measurement results achieved by the HT method remain relatively constant. When the noise level increases from 10% to 20%, the absolute height errors introduced by the centroid and FFT methods increase significantly, especially for the centroid method. In contrast, the errors introduced by the HT method only increase slightly as the noise increases. Therefore, the HT method is superior to the centroid and FFT methods in terms of noise immunity. It can also be noted that the centroid method seems to be more sensitive to random noise as it introduces more measurement errors at a higher noise level. This is because the superimposed random noise causes the simulated interferometric signal to be distorted and not perfectly symmetrical, resulting in a deviation of the obtained centroid from the desired envelope peak.

With a 20% random noise added, a comparison of simulation results obtained by different algorithms is shown in Table 1. The mean height values obtained by different algorithms do not differ significantly. However, the maximum standard deviation produced by the centroid method is 0.223 μm, which is much bigger than those of the FFT and HT methods. The FFT method requires the most computation time due to one forward FT and one inverse FT, while the centroid method greatly reduces the data processing steps and provides a high measurement speed. The HT method provides a higher calculation efficiency than the FFT method due to the simplification in convolution operation.

### 3.2. Effect of Scanning Interval

**Figure 5:**
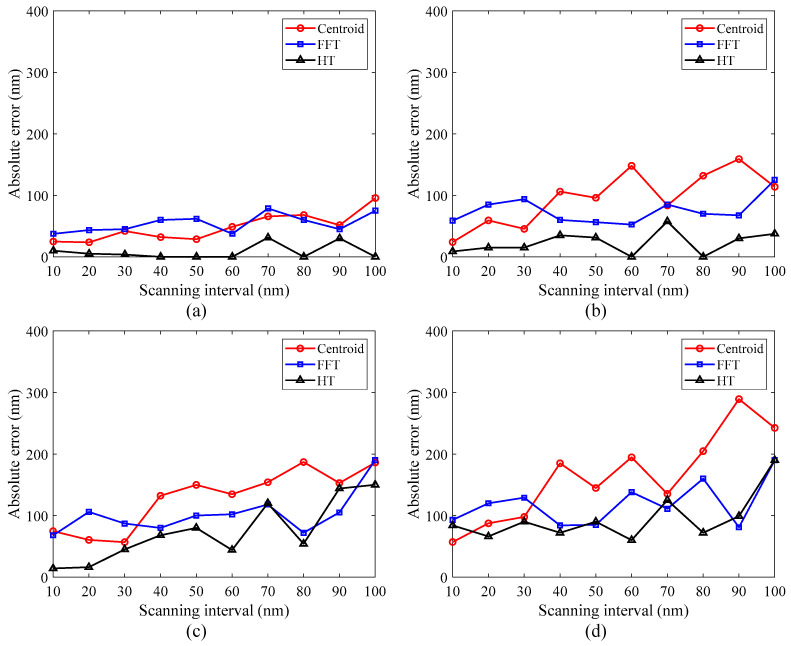
Comparison of errors introduced by the scanning interval under different noise levels: (**a**) 5% random noise, (**b**) 10% random noise, (**c**) 15% random noise, and (**d**) 20% random noise.

The scanning interval is an important parameter in VSI, which influences the measurement sensitivity and measurement efficiency. A smaller scanning interval means that the measurement system would collect more fringe images within the same scanning distance and hence require more calculation time. However, a larger scanning interval may not only lead to a larger positioning error of the scanner but also affect the smoothness of the reconstructed surface. The effect of the scanning interval on the measurement results was investigated under different noise levels. The relationship between the absolute errors and the scanning interval was obtained. As shown in Figure 5, with 5% random noise added, the errors vary slightly as the scanning interval increases from 10 nm to 100 nm. When 10%, 15%, and 20% random noise is added, respectively, the errors generally increase with the growing scanning interval, especially when the scanning interval exceeds 60 nm. It can also be seen that the results obtained by the centroid method are more susceptible to the scanning interval at a high noise level. In contrast, the errors introduced by the HT method have a relatively smaller increase with the scanning interval. Even if the results obtained by the FFT method fluctuate somewhat with the change in scanning interval, they show some similar trends with those of the HT method. Considering the measurement sensitivity and efficiency, the scanning interval of 30–50 nm is more appropriate in the actual measurement process. In addition to a smaller scanning interval, an appropriate interpolation technique is also important to improve the axial sensitivity that is limited by undersampling along the optical axis. It has been reported that the envelope peak curve can be corrected by using the Gaussian estimation and interpolation [30]. The interpolation technique based on second-order spline fitting can improve axial sensitivity, and post-filtering methods are also helpful to reduce measurement noise, providing a higher axial sensitivity [41].

## 4. Experimental Verification and Discussion

### 4.1. Experimental Setup

**Figure 6:**
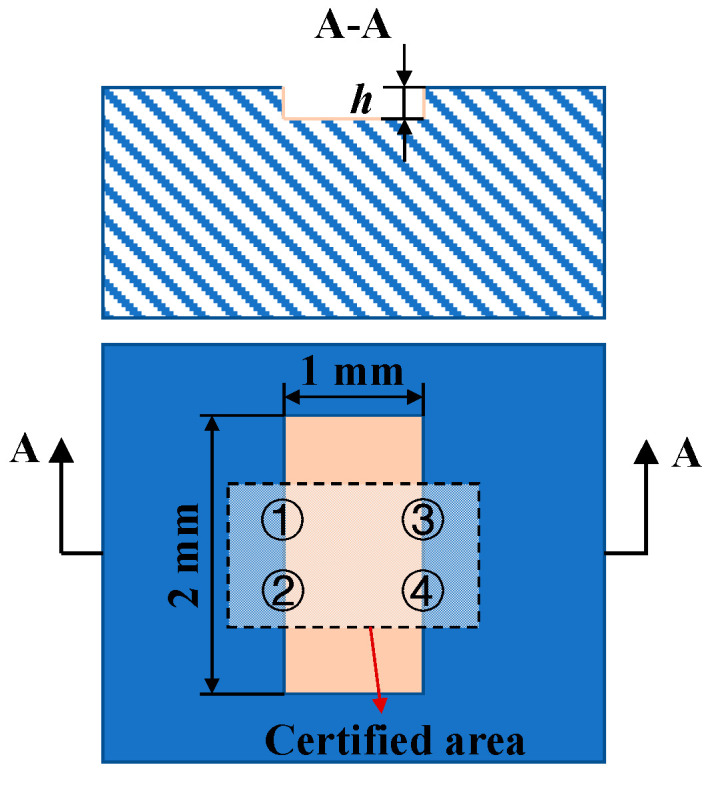
Cross section of step height standard.

To verify the simulation results, the experiment was carried out on a miniaturized white-light interferometer that was developed for on-machine surface measurement. The validity and accuracy of the measurement systems and related algorithms can be evaluated by measuring calibrated standards [42,43]. Two step height standards, 301–113–8UM (height = (7.347 ± 0.018) μm) and 301–113–50UM (height = (47.022 ± 0.048) μm) (Bruker Corporation) were measured to assess the performance of the three algorithms. The certified values of the step heights are traceable to the International System of Units (SI) by the Physikalisch-Technische Bundesanstalt (PTB). As shown in Figure 6, the negative step height feature is etched in the middle of the silicon chip to the nominal height and then is coated with a uniform 100 nm thick layer of chromium [44]. Due to the limitation of the field of view (FOV) of the objective, the whole step height feature cannot be obtained in one measurement. Alternatively, four measurement positions were selected to calculate the mean step height in the certified area, named as Positions 1, 2, 3, 4, respectively. The four corners of the rectangular certified area on the step height standard were selected as reference points. By recording the distance between the reference points and the measurement points, the same positions to be measured can be found even if the sample is mounted on different instruments.

**Figure 7:**
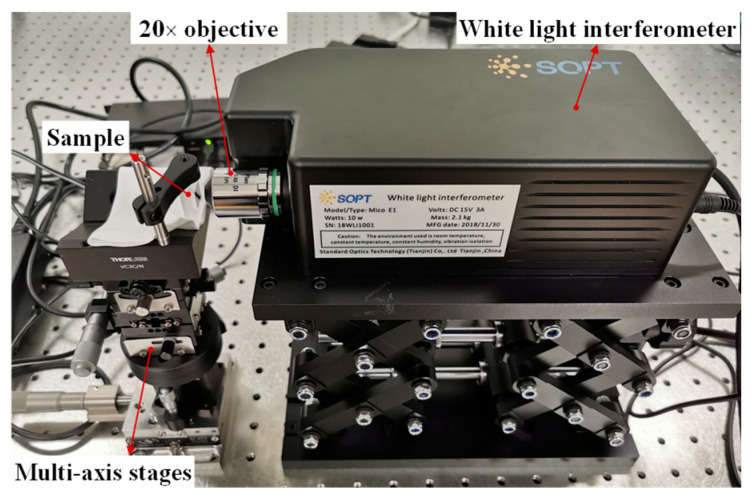
Experimental setup of step height measurements.

Figure 7 shows the experimental setup of step height measurements. The experimental apparatus was placed on a vibration-isolated optical table. The sample was mounted on a multi-axis stage. The white light interferometer was placed horizontally since this was more similar to the application scenario of on-machine measurement in ultra-precision machining. This compact interferometer is equipped with a 20× Mirau-type objective (403854, Olympus, Tokyo, Japan) having a numerical aperture (NA) of 0.4. The measurement system is illuminated by an LED-based light source (white light, output power: 1–5 W, working distance: 20–60 mm, maximum illumination: 240 lux, color temperature: 3000–6000 K), offering an FOV of 0.34 mm × 0.27 mm (640 pixels × 512 pixels) and 0.4 mm vertical scanning range. In measurement experiments, a series of white light interferograms were acquired by the CCD camera when the scanning interval was 30 nm. To verify the reliability of the measurement results, the step height standards were also measured by a commercial white-light interferometer (NPFLEX™ 3D Surface Metrology System, Bruker Corporation, Bill Rica, MA, USA), named as NPFLEX. A 20× Mirau-type objective (SN 681306–60, Nikon, Tokyo, Japan) with a NA of 0.4 was used, providing an FOV of 0.44 mm × 0.33 mm (1376 pixels × 1040 pixels) that was similar to that of the developed system.

### 4.2. Experimental Results

**Figure 8:**
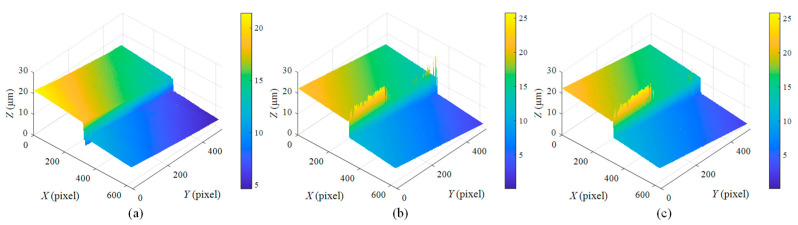
Reconstructed surface profile of 7.347 μm step height by using: (**a**) centroid method, (**b**) fast Fourier transform (FFT) method, and (**c**) Hilbert transform (HT) method.

Figure 8 shows the reconstructed surface profile of the 7.347 μm step height at Position 1 by using different algorithms. It can be seen that the raw reconstructed surfaces are tilted and there are measurement errors at the step edges. Although the centroid method can average out the noise at the step edge, the reconstructed surface profile is still slightly distorted. The FFT and HT methods present similar surface profiles where the upper surface and lower surface are both flat. However, severe noise can be observed at the step edges. Since surface reconstruction is seriously affected by the poor acquisitions of interferometric fringes at the step edges, the outliers in the raw measurement data have been removed under the same parameters.

**Figure 9:**
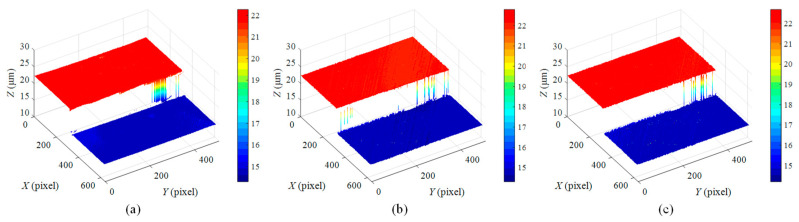
Surface profile of 7.347 μm step height reconstructed by using: (**a**) centroid method, (**b**) FFT method, (**c**) HT method.

In Figure 9, there are some missing points at the step edges, corresponding to the blank areas in the reconstructed profile, so a non-adequate step height artefact is presented.

**Figure 10:**
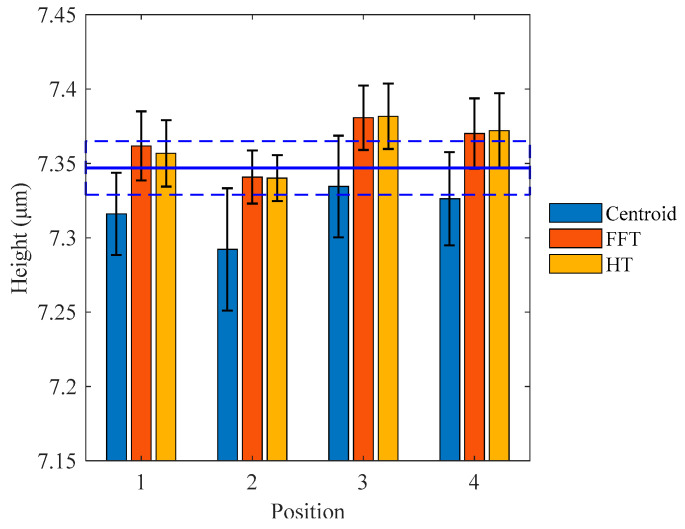
Comparison of mean heights and standard deviations of 7.347 μm step height at different positions.

The measurement process was repeated 8 times at each position within the certified area. Figure 10 shows the measurement results of the 7.347 μm step height at different positions. The blue solid line indicates the certified value, and the rectangular dashed box indicates the error bar of the certified height. It can be seen that all the error bars based on the standard deviation of 8 measurements for each algorithm can overlap with the error bar of the certified height. The mean height values obtained by the centroid method are below the certified value, while the FFT and HT methods yield similar results only at Position 2. The mean height values of Position 3 obtained by the three methods are larger than those of other positions. The maximum standard deviation is produced by the centroid method, and the measurement results obtained by the FFT and HT methods do not show a significant difference in terms of mean height and standard deviation.

**Figure 11:**
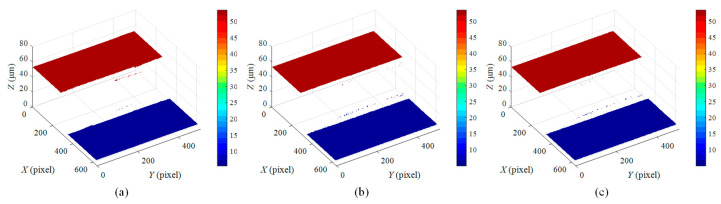
Surface profile of 47.022 μm step height reconstructed by using: (**a**) centroid method, (**b**) FFT method, (**c**) HT method.

Figure 11 shows the reconstructed surface profile of the 47.022 μm step height after removing the outliers. When the step height increases to 47.022 μm, more missing points appear at the step edges, but this issue does not affect the calculation of the step height.

**Figure 12:**
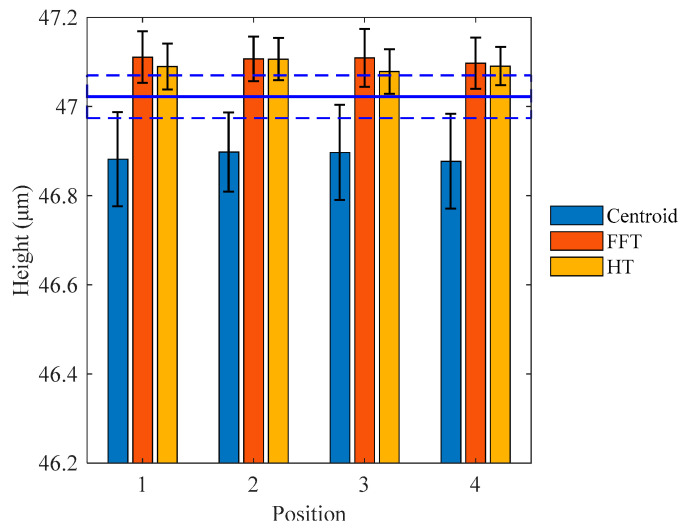
Comparison of mean heights and standard deviations of 47.022 μm step height at different positions.

Figure 12 shows the measurement results of the 47.022 μm step height at different positions. The mean height values achieved by the centroid method are markedly smaller than the certified value, while the algorithms based on the FFT and HT methods provide mean height values slightly larger than the certified value. The mean height values and standard deviations achieved by the FFT and HT methods are similar and closer to the certified value corresponding to the blue solid line.

Table 2 and Table 3 show the comparisons of measurement results obtained by different methods. For the 7.347 μm step height, the mean height value achieved by the HT method is 0.015 μm larger than the certified value, with a standard deviation of 0.027 μm. For the 47.022 μm step height, the mean height value achieved by the HT method is 0.070 μm larger than the certified value, with a standard deviation of 0.047 μm. Accordingly, the NPFLEX yields a mean height of 7.339 μm with a standard deviation of 0.005 μm for the 7.347 μm step height, and a mean height of 47.052 μm with a standard deviation of 0.023 μm for the 47.022 μm step height, respectively.

### 4.3. Discussion

**Figure 13:**
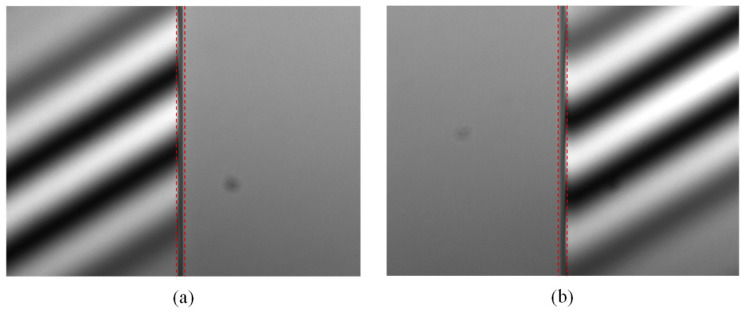
White-light interferograms generated on 7.347 μm step height: (**a**) upper surface, and (**b**) lower surface.

The reconstructed surface profiles shown in Figure 8 are seriously affected by the poor acquisitions of interferometric fringes at the step edges. This is likely attributable to the surface discontinuities. In this case, the coherence envelope obtained at the step edges would be distorted, and its peak position would shift [12,33]. This issue can be further explained by Figure 13, in which high-contrast white-light interferograms are generated both on the upper surface and lower surface, but no interferogram can be seen at the step edges. This is due to the limited NA of the objective that characterizes the spread of angles over which the reflected light is accepted by the objective. If an objective with a low NA is used to measure a surface with significant discontinuous features, the reflected lights from the discontinuous surface regions may not be within the range of the optical imaging system, and thus outliers may appear in the measured data. Consequently, the limited NA would cause reduced light reflection and poor SNR of the interferometric signal at the step edge.

The surface profile reconstructed by the centroid method is not flat enough as shown in Figure 9a and Figure 11a, because environmental disturbances and system noise may undermine the symmetry of the interferometric signals. The upper and lower surfaces reconstructed by the FFT and HT methods have a better flatness than those reconstructed by the centroid method because these two methods have better noise immunity. The centroid method offers high measurement speed because the centroid of the interference signal is utilized to estimate the peak position of the corresponding modulation function [17], effectively reducing the computational complexity. However, the step profile reconstructed through the centroid method is somewhat distorted, resulting in a larger height deviation. In contrast, the FFT method takes the most computation time due to its global character. The digitized fringe images need to be filtered to obtain the envelope function by two FT processes. The FFT and HT methods outperform the centroid method in terms of measurement accuracy. The HT method offers similar measurement results to the FFT method because they share a similar working principle. Moreover, the HT method is more competitive in terms of measurement speed due to the convolution operation, which allows a greater reduction in computation time than the FFT method. It should be noted that the measurement errors discussed here include not only the errors caused by the step heights and algorithms used, but also the errors that are attributed to the positioning error of the PZT, mechanical vibration, and the thermal drift of the measurement system.

**Figure 14:**
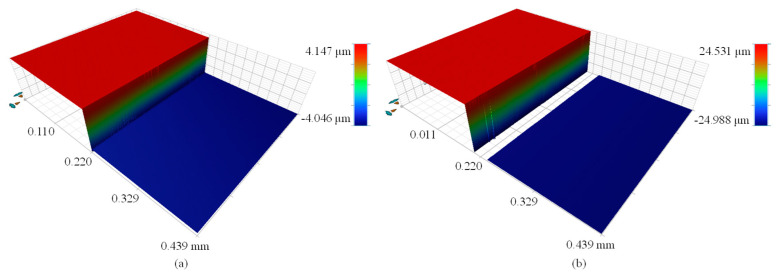
Step profile measured by the NPFLEX (VSI mode): (**a**) 7.347 μm step height, (**b**) 47.022 μm step height.

Figure 14 shows the step profile measured by the NPFLEX. When measuring the 7.347 μm step height, the step profile is well presented with little noise and very few missing points appear at the step edges. When the step height increases to 47.022 μm, there are more missing points at the step edges, and the upper surface and lower surface are completely separated, which agrees with the measurement performance of the developed interferometer. The total measurement time of the NPFLEX, including fringe acquisition and data processing, is less than that of the developed interferometer, regardless of using any one of the three algorithms. However, the details of the hardware and software of this commercial instrument are not fully disclosed, so the measurement time is not rigorous for comparison. In addition, the measurement errors of the developed interferometer are almost twice as high as that of the NPFLEX. The underlying causes of the difference in measurement performance of these two instruments are complex. A remarkable feature of the developed interferometer is that it can be installed on a machine motion stage and can perform the on-machine surface measurement but at the expense of some hardware performance. On the other hand, even if the FOV of the developed interferometer is close to that of the NPFLEX, the number of pixel arrays of the former is smaller than that of the latter. Since the fringe envelope obtained on each pixel of the sensor can be used to calculate the surface height, more pixel arrays typically allow smaller details to be resolved [45]. Hence, the NPFLEX can produce better surface topography information and higher measurement accuracy.

In conclusion, the experimental results obtained by the developed interferometer are in good agreement with the simulation results and are also consistent with those obtained by the NPFLEX, which proves the reliability of the presented measurement results. The performance of the developed interferometer and surface reconstruction algorithms for on-machine metrology will be further investigated in future work.

## 5. Conclusions

This paper presents a comparative study of envelope detection techniques in VSI. Three kinds of fringe analysis algorithms based on the centroid method, FFT, and HT methods are discussed. The simulation results of white-light interferograms show that the centroid method provides a high measurement speed but can only offer acceptable measurement accuracy at a low noise level. The HT method is superior to the centroid and FFT methods in terms of noise immunity. Moreover, the measurement errors increase markedly with the increase of the scanning interval at a high noise level, especially when the scanning interval exceeds 60 nm. Practical measurement experiments of step height standards were carried out on a miniaturized white-light interferometer that was developed for on-machine surface measurement. The experimental results show that the measurement errors obtained by the FFT- and HT-based algorithms are less than 100 nm when measuring a 47.022 μm step height. Even if the FFT and HT methods offer similar performance and outperform the centroid method in terms of measurement accuracy, the measurement speed of the HT method is nearly twice as fast as the FT method. The experimental results obtained by the developed interferometer match well the simulation results and are also consistent with the measurement results obtained by a commercial white-light interferometer. In future work, the results of this study will be applied to on-machine surface measurement. It would be interesting to verify the performance of different surface reconstruction algorithms in a manufacturing environment.

## Figures and Tables

**Table 1:** Comparison of simulation results obtained by the three algorithms.

Method	Theoretical Height (μm)	Mean Height (μm)	Standard Deviation (μm)	Computation Time (μs)
Centroid	30	30.063	0.223	20.8
Fast Fourier transform (FFT)	30	30.027	0.090	1281.8
Hilbert transform (HT)	30	29.970	0.028	215.5

**Table 2:** Measurement results of 7.347 μm step height.

Method	Mean Height (μm)	Standard Deviation (μm)	Measurement Error (μm)	Computation Time (s)
Centroid	7.317	0.036	0.030	8.728
FFT	7.364	0.026	0.017	69.515
HT	7.362	0.027	0.015	35.256
NPFLEX	7.339	0.005	0.008	-

**Table 3:** Measurement results of 47.022 μm step height.

Method	Mean Height (μm)	Standard Deviation (μm)	Measurement Error (μm)	Computation Time (s)
Centroid	46.889	0.097	0.133	31.268
FFT	47.106	0.055	0.084	261.094
HT	47.092	0.047	0.070	126.866
NPFLEX	47.052	0.023	0.030	-

## Data Availability

Data sharing is not applicable to this article.
